# Integrated Drought-Response Profiles Reveal Genotypic Variation Among Commercial Oil Palm (*Elaeis guineensis* Jacq.) Genotypes

**DOI:** 10.3390/plants15203165

**Published:** 2026-10-16

**Authors:** Sunya Nuanlaong, Chandrasekhar Manikala, Thanet Khomphet, Azzreena Mohamad Azzeme, Potjamarn Suraninpong

**Affiliations:** 1 School of Agricultural Technology and Food Industry, Walailak University, Nakhon Si Thammarat 80161, Thailand; sunyaton@gmail.com (S.N.); chandrasekharmanikala003@gmail.com (C.M.); thanet.kh@wu.ac.th (T.K.); 2 Faculty of Biotechnology and Biomolecular Sciences, Universiti Putra Malaysia, Serdang 43400, Malaysia; azzreena@upm.edu.my; 3 Herbology Research Center, Walailak University, Nakhon Si Thammarat 80161, Thailand

**Keywords:** *Elaeis guineensis*, water withholding, genotypic variation, stomatal traits, carbohydrate responses, multivariate analysis

## Abstract

Drought stress significantly limits oil palm productivity, highlighting the need to characterize integrated responses to water deficit. This study evaluated morphological, stomatal, anatomical, and biochemical responses of ten commercial oil palm genotypes under well-watered and water-withholding conditions for 45 days. Quantitative analyses focused on 0–30 days after treatment (DAT), while 45 DAT represented a qualitative endpoint of severe injury. Water withholding restricted vegetative growth and altered stomatal characteristics, chlorophyll, soluble sugar, and starch contents, with responses varying among genotypes. Qualitative observations also revealed changes in bulliform cell morphology. Correlation analysis based on Δ30–0 values showed contrasting trait associations between water regimes. Principal component analysis of genotype-level relative responses at 30 DAT, based on five traits with complete data (CW, FN, TC, SC, and ST), showed that PC1 and PC2 explained 52.10% and 35.11% of the total variation, respectively, accounting for 87.21% cumulatively. Hierarchical cluster analysis of the same standardized relative-response dataset separated the ten genotypes into three groups with contrasting multivariate response profiles. These findings demonstrate substantial genotype-dependent variation in integrated responses to water withholding and indicate that combining multiple complementary traits can distinguish contrasting response profiles among young oil palm genotypes. This multi-trait approach may provide a useful basis for subsequent drought evaluation and breeding, although further validation using direct measurements of soil and plant water status, recovery responses, and field-based drought conditions is required.

## 1. Introduction

Drought is one of the major environmental constraints affecting agricultural productivity worldwide. Climate change is expected to intensify agricultural water scarcity across many croplands, increasing the risk of water limitation for crop production [1]. Drought-induced water deficits can substantially impair plant growth and development [2]. Under water-deficit conditions, plants undergo coordinated morphological, anatomical, physiological, and biochemical adjustments to reduce water loss and maintain cellular function. These responses include stomatal regulation, osmotic adjustment through compatible solute accumulation, and modifications in leaf morphology and metabolism [1,3]. The magnitude and coordination of these responses vary among genotypes and may contribute to genotypic differences under water limitation [4].

Oil palm (*Elaeis guineensis* Jacq.) is one of the world’s most important oil-producing crops and requires adequate water availability for optimal growth and productivity [5]. Drought stress can adversely affect oil palm performance by restricting vegetative and reproductive development and reducing photosynthetic activity [6,7]. Water limitation can also affect reproductive development by altering sex determination and increasing inflorescence abortion, with potential consequences for fruit bunch formation and yield [8,9]. Such reproductive effects, together with reductions in vegetative growth and photosynthetic performance, may ultimately constrain oil palm productivity under increasingly variable climatic conditions [10].

Drought responses in oil palm involve changes in multiple traits, including vegetative growth, stomatal characteristics, photosynthetic pigments, and carbohydrate metabolism [4,11]. Growth reduction may occur together with stomatal adjustment and changes in carbon allocation under limited water availability [12,13]. Soluble carbohydrates can serve as metabolic substrates and contribute to osmotic adjustment and cellular protection during dehydration [13,14]. Leaf anatomical adjustments may also accompany responses to water limitation. In monocotyledonous plants, changes in bulliform cell turgor are associated with leaf rolling, which can reduce exposed leaf area and transpirational water loss [15]. Molecular studies in oil palm further indicate that drought responses involve changes in stress-related genes and metabolic pathways, including carbohydrate-associated processes [16]. Because these responses may occur simultaneously and differ among genotypes, evaluating multiple complementary traits may provide a more comprehensive characterization of drought-response patterns than considering individual traits alone. For oil palm breeding, such an integrated evaluation at the seedling stage may help differentiate contrasting response profiles among genotypes and identify germplasm for subsequent drought evaluation.

Although individual drought responses in oil palm have been investigated, integrated comparisons of morphological, stomatal, anatomical, and biochemical responses across commercial genotypes remain limited. Moreover, the potential utility of these complementary traits for distinguishing contrasting response patterns at the seedling stage has received relatively limited attention. Therefore, this study evaluated ten commercial oil palm genotypes under an imposed water-withholding regime, focusing on quantitative responses during 0–30 days after treatment (DAT), with 45 DAT retained as a qualitative endpoint of severe injury. Morphological, stomatal, and biochemical traits, together with qualitative observations of bulliform cell morphology, were evaluated using correlation and multivariate analyses to characterize genotype-dependent response patterns. We hypothesized that commercial genotypes would exhibit contrasting and coordinated responses to water withholding and that integrating multiple response traits could help distinguish contrasting response profiles among young oil palm genotypes for subsequent drought evaluation and breeding.

## 2. Results

### 2.1. Morphological and Vegetative Growth Responses

Morphological and vegetative growth traits were significantly affected by genotype (G), water regime (W), and time (T) (Table 1). Significant main effects of genotype, water regime, and time were detected for canopy width (CW), frond number (FN), leaflet number (LLN), and leaf area (LA) (all *p* < 0.001). The G × W interaction was significant for FN (*p* < 0.001) and LA (*p* < 0.01), but not for CW or LLN. W × T interactions were significant for all four traits (*p* < 0.001), whereas G × W × T interactions were significant for CW, FN, and LA (*p* < 0.001), but not for LLN.

**Figure 1:**
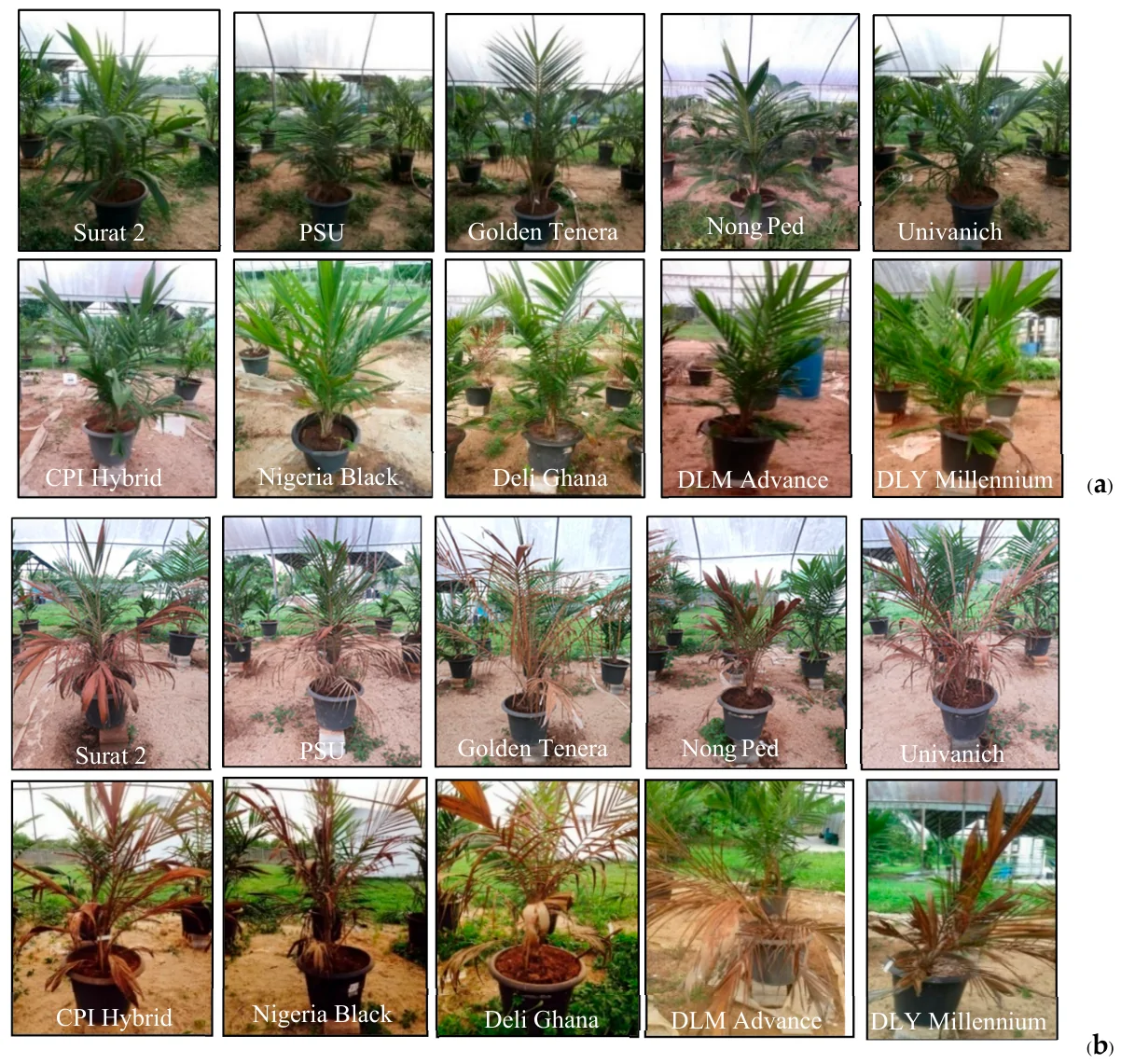
Morphological appearance of ten oil palm genotypes after 45 days of treatment. (**a**) Well-watered plants and (**b**) water-withholding-treated plants showing severe injury symptoms, including leaf desiccation, necrosis, and spear leaf curling. Images at 45 DAT are presented as a qualitative endpoint of severe injury and were not included in the primary quantitative analyses.

Temporal changes demonstrated contrasting growth patterns between water regimes (Table S1). Under well-watered conditions, vegetative growth was generally maintained or increased, whereas water withholding progressively restricted growth over 0–30 DAT. Changes from baseline to 30 DAT (Δ30–0) were significantly affected by genotype and water regime, with significant G × W interactions detected for CW and FN (Table S2). By 45 DAT, water-withholding-treated plants exhibited pronounced leaf desiccation, necrosis, and spear leaf curling (Figure 1), representing a qualitative endpoint of severe injury beyond the primary 0–30 DAT quantitative assessment period.

### 2.2. Stomatal and Guard-Cell Responses

Stomatal and guard-cell traits were significantly affected by genotype, water regime, and time (Table 2). Significant main effects of genotype, water regime, and time were detected for stomatal pore width (SPW), stomatal pore length (SPL), guard-cell width (GCW), guard-cell length (GCL), and stomatal density (SN) (all *p* < 0.001). All two-way and three-way interactions were also significant for all five traits (all *p* < 0.001). For changes from baseline to 30 DAT (Δ30–0), significant genotype, water-regime, and G × W effects were detected for SN, stomatal pore size index (SPSI), and guard-cell pair size index (GCPSI) (Table S2). The corresponding baseline absolute values of the stomatal and guard-cell traits at 0 DAT are provided in Table S3.

### 2.3. Bulliform Cell Responses

**Figure 2:**
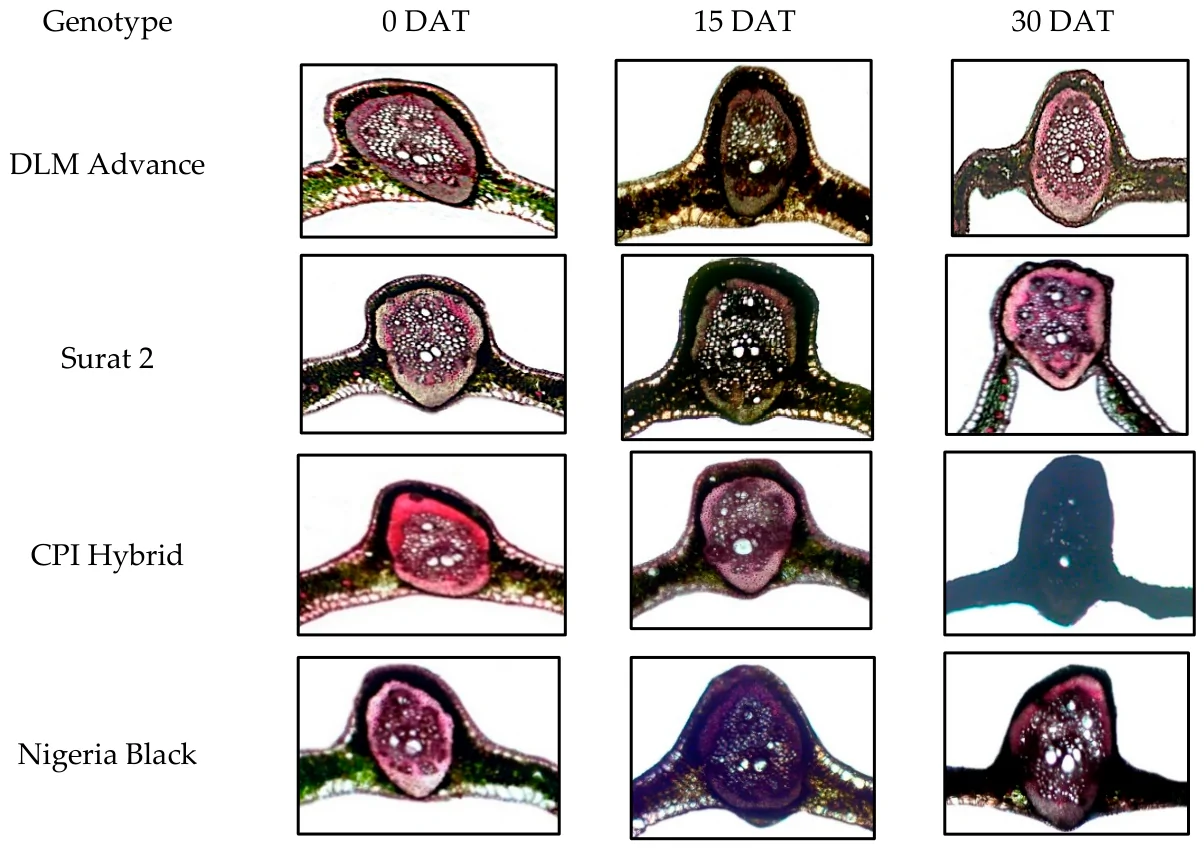
Representative changes in bulliform cell morphology of four oil palm genotypes under the water-withholding treatments at 0, 15, and 30 days after treatment (DAT). All images were captured at the same magnification (5×).

Qualitative observations showed changes in bulliform cell morphology under the water-withholding treatment at 15 and 30 DAT compared with 0 DAT across the ten genotypes (Figure S1). Representative changes in four genotypes are shown in Figure 2. These observations were qualitative and were not subjected to statistical analysis.

### 2.4. Biochemical Responses

Biochemical traits showed significant effects of genotype, water regime, and time, although the patterns differed among traits (Table 3). Genotype significantly affected all traits (*p* < 0.001), whereas water regime affected soluble sugar content (SC) (*p* < 0.05) and starch content (ST) (*p* < 0.001), but not chlorophyll a (Chl *a*), chlorophyll b (Chl *b*), or total chlorophyll (TC). Significant G × W interactions were detected for Chl *a* (*p* < 0.01), Chl *b*, TC, and ST (all *p* < 0.001), but not SC. Time significantly affected Chl *b* and ST (*p* < 0.001), TC (*p* < 0.01), and SC (*p* < 0.001), but not Chl *a*. G × T interactions were significant for Chl *a*, Chl *b*, SC, and ST, whereas G × W × T interactions were significant for Chl *a*, Chl *b*, and TC. Baseline absolute values of the biochemical traits at 0 DAT are provided in Table S3.

Temporal changes in biochemical traits from 0 to 30 DAT varied among genotypes under the water-withholding treatment (Table S4). TC decreased in most genotypes, with the largest decline observed in Nong Ped (ΔTC = −0.862), whereas CPI Hybrid showed an increase (ΔTC = 0.255). Nigeria Black showed only a small decrease (ΔTC = −0.061). SC responses were variable, with the largest increase observed in Deli Ghana (ΔSC = 0.080). ST increased in Surat 2, PSU, Golden Tenera, Nong Ped, Univanich, and Nigeria Black, but decreased in CPI Hybrid, Deli Ghana, DLM Advance, and DLY Millennium.

### 2.5. Correlation Analysis

**Figure 3:**
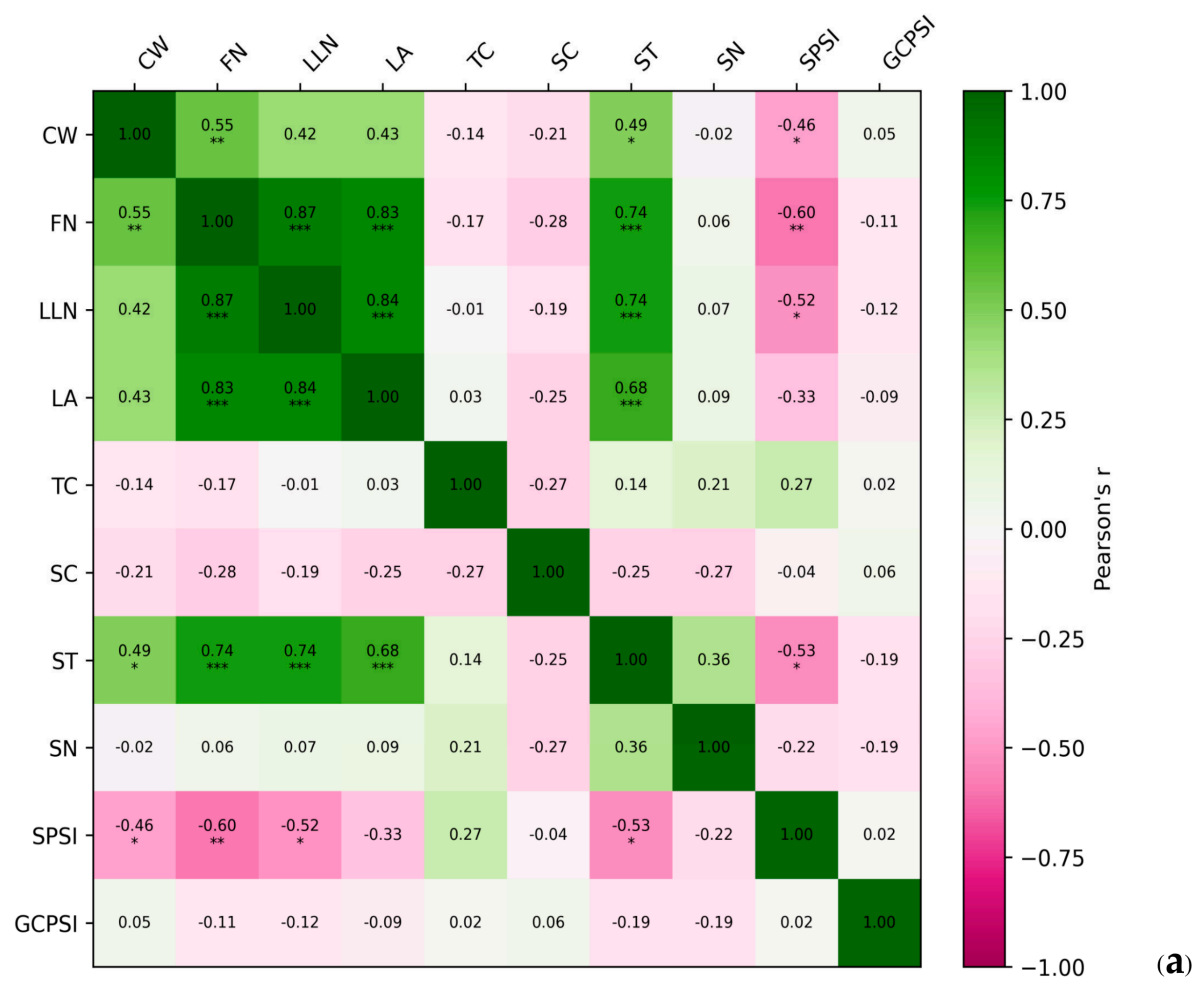

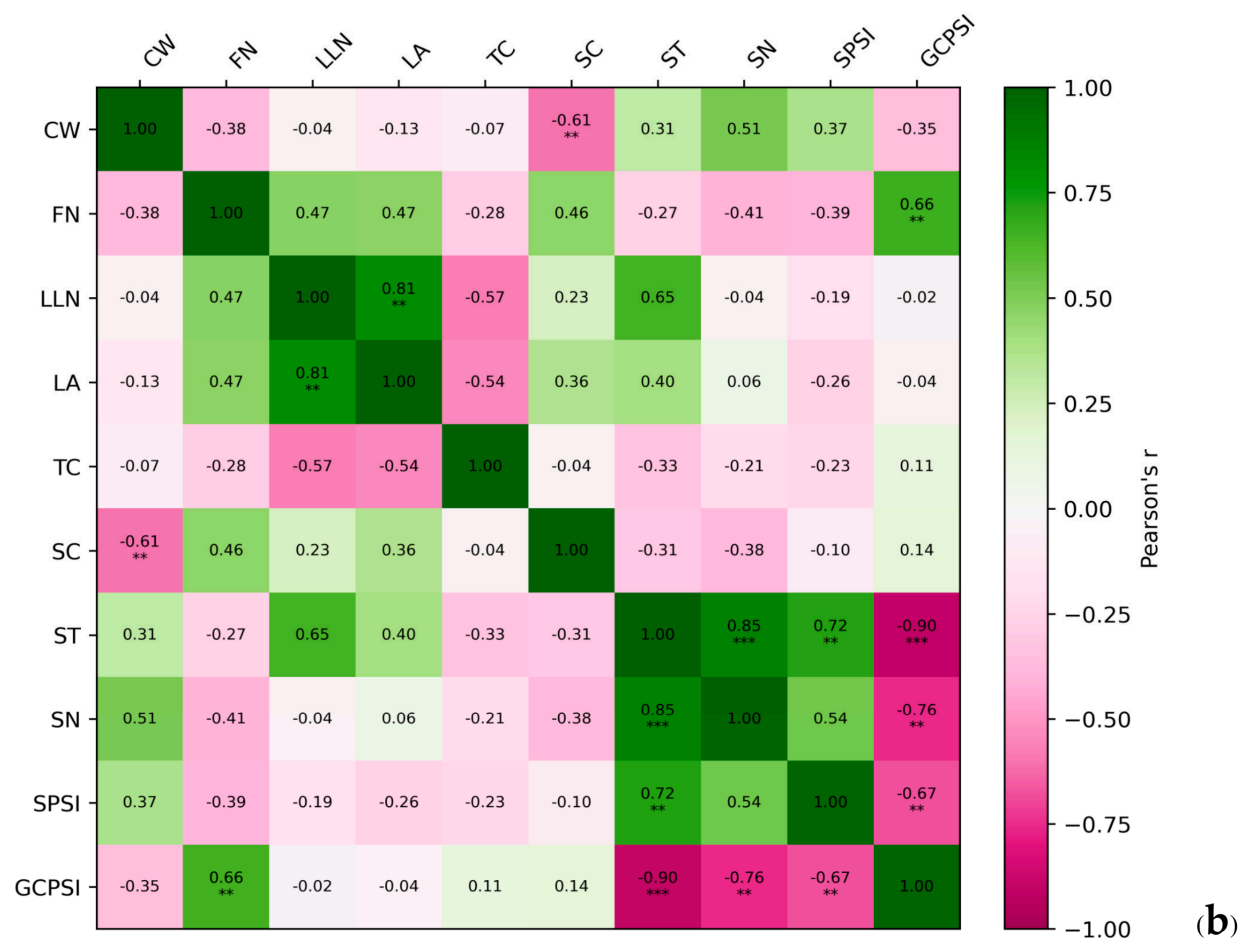
Pearson correlation matrices among morphological, biochemical, and stomatal traits based on changes from 0 to 30 days after treatment (Δ30–0) under (**a**) well-watered and (**b**) water-withholding conditions. Correlations were calculated separately for each water regime using individual biological replicates and pairwise complete observations. Missing measurements were not imputed. Positive and negative correlations are represented by the color scale. Asterisks indicate correlations significant after Benjamini–Hochberg false-discovery-rate (FDR) adjustment: *, FDR-adjusted *p* < 0.05; **, FDR-adjusted *p* < 0.01; and ***, FDR-adjusted *p* < 0.001.

Pearson’s correlation analysis revealed distinct trait associations between well-watered and water-withholding conditions (Figure 3; Table S5). After Benjamini–Hochberg false-discovery-rate (FDR) adjustment, strong positive correlations under well-watered conditions were observed among vegetative traits, including FN–LLN (*r* = 0.87), LLN–LA (*r* = 0.84), and FN–LA (*r* = 0.83; all FDR-adjusted *p* < 0.001). ST was positively correlated with FN (*r* = 0.74), LLN (*r* = 0.74), and LA (*r* = 0.68; all FDR-adjusted *p* < 0.001), whereas SPSI was negatively correlated with FN (*r* = −0.60, FDR-adjusted *p* < 0.01), LLN (*r* = −0.52, FDR-adjusted *p* < 0.05), and ST (*r* = −0.53, FDR-adjusted *p* < 0.05).

Under water deficit, LLN remained strongly correlated with LA (*r* = 0.81, FDR-adjusted *p* < 0.01), whereas CW was negatively correlated with SC (*r* = −0.61, FDR-adjusted *p* < 0.01). ST was strongly positively correlated with SN (*r* = 0.85, FDR-adjusted *p* < 0.001) and SPSI (*r* = 0.72, FDR-adjusted *p* < 0.01), but negatively correlated with GCPSI (*r* = −0.90, FDR-adjusted *p* < 0.001). SN was negatively correlated with GCPSI (*r* = −0.76, FDR-adjusted *p* < 0.01), and SPSI was also negatively correlated with GCPSI (*r* = −0.67, FDR-adjusted *p* < 0.01).

### 2.6. Principal Component Analysis

**Figure 4:**
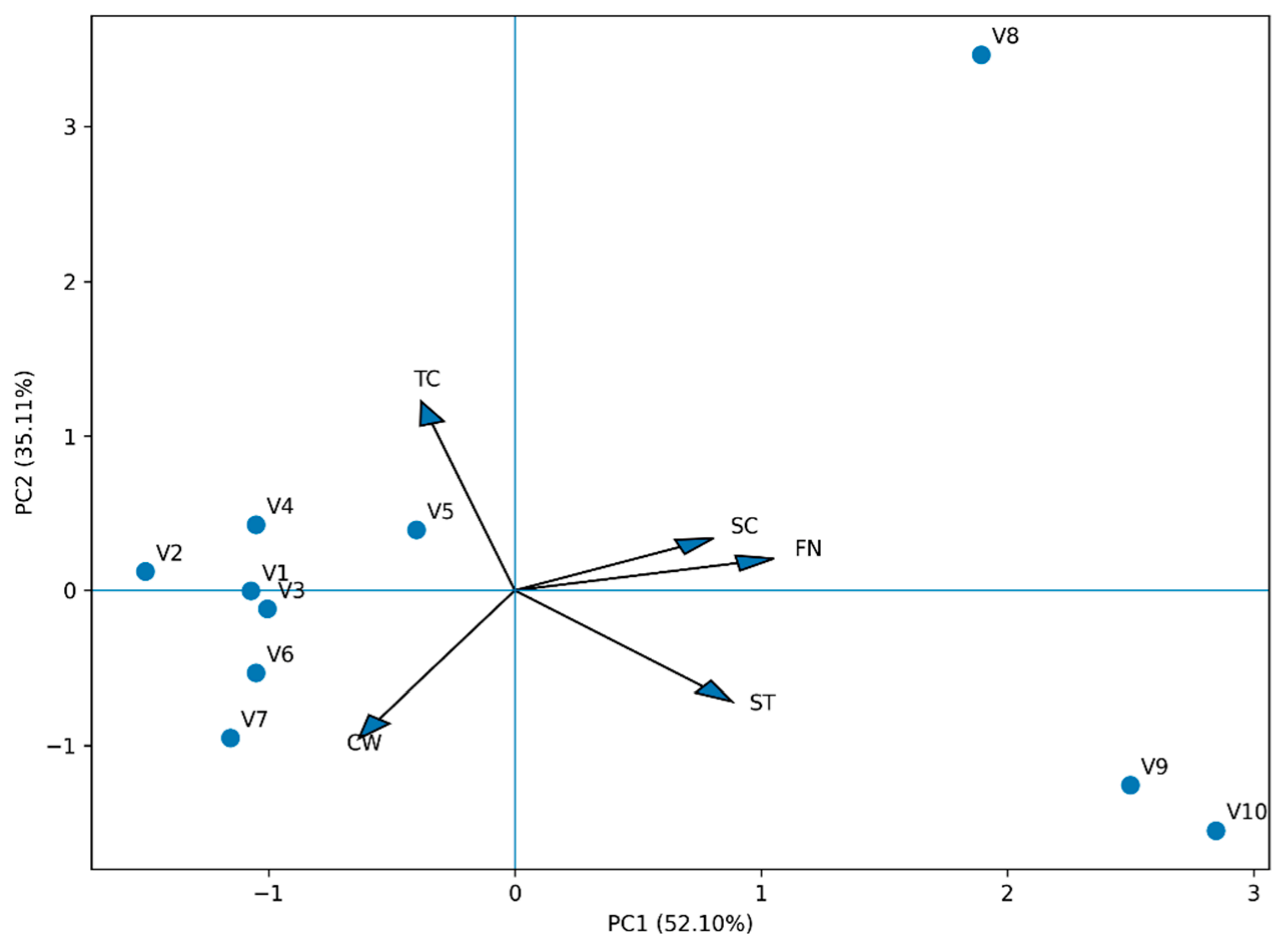
Principal component analysis (PCA) of Z-score-standardized relative responses to water withholding at 30 days after treatment (DAT) in ten oil palm genotypes. Relative responses were calculated as (mean water-withholding value at 30 DAT/mean well-watered value at 30 DAT) × 100 for the five traits with complete genotype-level data: canopy width (CW), frond number (FN), total chlorophyll (TC), soluble sugar content (SC), and starch content (ST). PC1 and PC2 explained 52.10% and 35.11% of the total variation, respectively, accounting for 87.21% cumulatively. Trait vectors indicate the direction and relative contribution of the five traits to the principal components. Genotype codes are as follows: V1, Surat 2; V2, PSU; V3, Golden Tenera; V4, Nong Ped; V5, Univanich; V6, Nigeria Black; V7, CPI Hybrid; V8, Deli Ghana; V9, DLM Advance; and V10, DLY Millennium.

Principal component analysis (PCA) based on the standardized relative responses of the five traits with complete genotype-level data (CW, FN, TC, SC, and ST) showed that PC1 and PC2 explained 52.10% and 35.11% of the total variation, respectively, accounting for 87.21% cumulatively (Figure 4; Table S6).

### 2.7. Hierarchical Cluster Analysis

**Figure 5:**
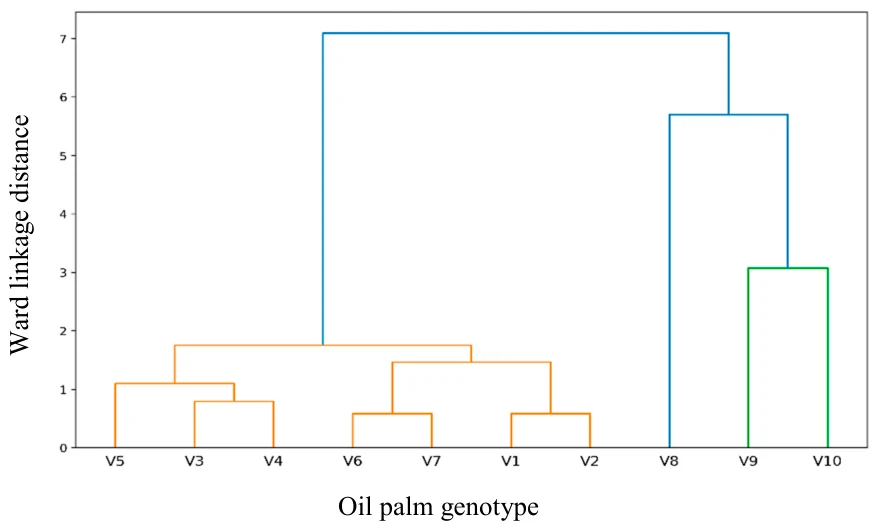
Hierarchical cluster analysis (HCA) of Z-score-standardized relative responses to water withholding at 30 days after treatment (DAT) in ten oil palm genotypes. Relative responses were calculated as (mean water-withholding value at 30 DAT/mean well-watered value at 30 DAT) × 100 for the five traits with complete genotype-level data: canopy width (CW), frond number (FN), total chlorophyll (TC), soluble sugar content (SC), and starch content (ST). Clustering was performed using Euclidean distance and Ward’s minimum variance method. Genotype codes are as follows: V1, Surat 2; V2, PSU; V3, Golden Tenera; V4, Nong Ped; V5, Univanich; V6, Nigeria Black; V7, CPI Hybrid; V8, Deli Ghana; V9, DLM Advance; and V10, DLY Millennium.

Hierarchical cluster analysis based on the same standardized relative-response dataset separated the ten genotypes into three clusters (Figure 5; Table S7). Cluster 1 comprised Surat 2 (V1), PSU (V2), Golden Tenera (V3), Nong Ped (V4), Univanich (V5), Nigeria Black (V6), and CPI Hybrid (V7). Cluster 2 comprised DLM Advance (V9) and DLY Millennium (V10), whereas Deli Ghana (V8) formed a distinct third cluster.

## 3. Discussion

Commercial oil palm genotypes exhibited contrasting morphological, stomatal, anatomical, and biochemical responses to water withholding, indicating substantial genotype-dependent variation in response patterns. This finding is consistent with previous evidence of differential drought responses among oil palm genotypes [4].

Water withholding progressively restricted vegetative growth, with the magnitude and temporal patterns differing among genotypes. Significant genotype × water regime × time interactions for CW, FN, and LA further demonstrated genotype-dependent growth responses. Reductions in vegetative growth under water stress have similarly been reported in oil palm seedlings [17]. Reduced canopy development and leaf area may reflect restricted cell expansion and biomass accumulation under limited water availability. Previous studies have also demonstrated variation in growth responses to water limitation among oil palm progenies and commercial genotypes [4,18,19,20], supporting the genotype-dependent patterns observed in the present study.

A further limitation concerns the use of the third frond from the top as the standardized sampling position. Although this approach reduced variation associated with canopy position, the same individual frond was not followed throughout the experiment. Because frond emergence and development can be affected by water limitation [5,21], variation in the developmental age of sampled fronds cannot be excluded. Consequently, some variation in leaf morphological, anatomical, stomatal, and chlorophyll traits may reflect differences in leaf developmental stage in addition to responses to water withholding [22,23]. Nevertheless, the consistent sampling criterion was applied to all plants and sampling times, and the results are therefore interpreted as integrated response patterns under the imposed water-withholding regime rather than as responses attributable solely to water withholding independently of leaf developmental stage.

Stomatal responses also varied markedly among genotypes under progressive water withholding, as reflected by significant genotype × water regime × time interactions. Changes in stomatal and guard-cell characteristics are important components of plant responses to declining water availability and may contribute to the regulation of gas exchange and transpirational water loss [3]. The contrasting magnitude and direction of these changes among genotypes indicate that stomatal adjustment was dynamic and genotype-dependent rather than a uniform response to water withholding, consistent with previous observations in oil palm under drought [4].

Qualitative anatomical observations revealed changes in bulliform cell morphology under water withholding, which became more apparent by 30 DAT. Bulliform cells are associated with leaf folding and rolling in monocots, and changes in their hydration and turgor may modify leaf exposure under water-limited conditions [15,24]. The observed morphological changes suggest that bulliform cells may be involved in the anatomical response of oil palm leaves to water withholding. However, quantitative anatomical and physiological measurements are needed to determine their contribution to genotypic variation in responses to water limitation.

Chlorophyll responses revealed genotype-dependent adjustments to water-withholding. Although water regime had no significant overall effect on Chl *a*, Chl *b*, or TC, significant genotype × water regime interactions indicated contrasting pigment responses among genotypes. Changes in chlorophyll content under drought have also been reported in oil palm seedlings, reflecting physiological adjustments under water-limited conditions [11]. The genotype-dependent pattern observed in the present study is also consistent with previous reports of contrasting physiological responses to water deficit among oil palm genotypes [4,7]. Because chlorophyll concentration does not directly measure photosynthetic activity, pigment retention should be interpreted as differential maintenance of photosynthetic pigments rather than evidence of sustained photosynthetic performance.

Carbohydrate responses also differed among genotypes. Water regime significantly affected both SC and ST, whereas the genotype × water regime interaction was significant only for ST, indicating stronger genotype dependence in starch responses. Over 0–30 DAT, ST increased in some genotypes but declined in others, while SC responses were more variable. Soluble sugars can contribute to osmotic adjustment, cellular protection, and metabolic maintenance during water deficit, whereas starch represents an important carbohydrate reserve whose allocation and utilization may change under drought [12,14]. Previous studies in oil palm have similarly reported drought-induced adjustments in soluble sugars and carbon metabolism, with responses differing among genetic materials [11,25]. These contrasting responses may reflect genotype-dependent differences in carbohydrate adjustment to water limitation [26]. Although molecular responses were not examined in the present study, transcriptomic studies have also demonstrated drought-responsive molecular and metabolic changes in oil palm [16].

Correlation analysis revealed marked shifts in trait associations under the water-withholding treatment. Under well-watered conditions, strong positive correlations among FN, LLN, and LA were consistent with coordinated vegetative development, while their positive associations with ST indicated relationships between vegetative growth and carbohydrate status. Under water withholding, associations shifted toward carbohydrate–stomatal relationships, particularly the positive correlations of ST with SN and SPSI and its negative correlation with GCPSI. These patterns indicate a reorganization of relationships among growth, stomatal, and carbohydrate-related responses under water withholding. Such associations are physiologically plausible because starch and sugar metabolism in guard cells is closely linked to osmotic regulation and stomatal movement [27,28].

PCA further supported this integrated response pattern, with the first two components explaining 87.21% of the total variation (PC1 = 52.10% and PC2 = 35.11%). PC1 was mainly associated with FN, ST, and SC, whereas PC2 was characterized primarily by positive loading of TC and negative loadings of CW and ST. The distribution of genotypes across the first two components therefore reflected coordinated variation in vegetative and biochemical responses rather than variation in a single trait. Similar multivariate approaches integrating multiple drought-responsive traits have been used to distinguish contrasting response profiles among oil palm cultivars and progenies and in other crop species [4,19,29].

HCA based on the standardized relative responses at 30 DAT separated the ten genotypes into three multivariate response groups: Surat 2, PSU, Golden Tenera, Nong Ped, Univanich, Nigeria Black, and CPI Hybrid; DLM Advance and DLY Millennium; and Deli Ghana. These groupings represent similarities in integrated relative responses to water withholding rather than discrete levels of drought tolerance. Similar multivariate approaches have been used in other crops to distinguish genotypes with contrasting drought-response patterns based on multiple traits, including relative trait responses analyzed by hierarchical clustering in wheat and coordinated morpho-physiological responses in soybean [29,30]. Interestingly, some clustering patterns appeared to correspond partly with parental background. DLM Advance and DLY Millennium, both derived from Deli × La Mé crosses and previously classified as drought-tolerant commercial materials, clustered together, suggesting that shared genetic background may contribute to similarities in their early water-withholding response profiles [31]. However, Surat 2, which also has a Deli × La Mé background and was previously classified as drought tolerant, clustered separately from DLM Advance and DLY Millennium, together with PSU, Golden Tenera, Nong Ped, Univanich, Nigeria Black, and CPI Hybrid. Previous molecular evidence likewise showed variation among La Mé-derived materials, with the CMO-associated genotype detected in Surat Thani 2 but not in DLM Advance or DLY Millennium [31]. These contrasting clustering patterns suggest that parental background alone may not fully explain the integrated response to water withholding, and that differences among parental sources and hybrid combinations may also contribute to the observed response patterns. Moreover, the present study evaluated 11-month-old seedlings during the first 30 days of water withholding, whereas established drought-tolerance classifications of commercial materials reflect broader genetic and agronomic performance. The three clusters identified here should therefore be interpreted as contrasting early-stage multivariate relative-response profiles under the imposed water-withholding treatment, rather than direct classifications of drought tolerance.

Oil palm seedlings exhibited coordinated morphological, stomatal, anatomical, and biochemical responses to water withholding, with substantial variation among commercial genotypes. Correlation and multivariate analyses revealed shifts in trait relationships and distinct genotype response profiles, indicating that no single trait adequately characterized responses to the imposed treatment across genotypes. Integrating changes in vegetative growth, stomatal characteristics, chlorophyll status, and carbohydrate responses may therefore provide a more comprehensive framework for preliminary characterization of contrasting response patterns among commercial germplasm for subsequent drought evaluation. However, these traits should be regarded as candidate indicators for preliminary screening rather than definitive markers of drought tolerance. However, these traits should be regarded as candidate indicators for preliminary screening rather than definitive markers of drought tolerance.

The greenhouse conditions, seedling developmental stage, relatively short analytical period, limited biological replication (n = 3 plants per genotype × water-regime combination), and lack of direct measurements of soil moisture and plant water status limit the interpretation and extrapolation of the present findings. In particular, because genotypes may differ in water use, the intensity and temporal development of water deficit may not have been identical among genotypes under the water-withholding treatment. As injury progressed, severe wilting and tissue deterioration also prevented reliable measurement of some traits in some plants, particularly when a frond meeting the predefined sampling criterion could no longer be reliably identified. These unavailable measurements were treated as missing rather than as biological zeros. Although differences in the timing of measurement loss may reflect variation in injury progression among genotypes, the present experimental design does not allow such missingness to be interpreted independently as evidence of drought susceptibility. The observed differences should therefore be interpreted as genotype-dependent responses to the imposed treatment rather than responses to an identical quantified level of drought stress. Light intensity (PAR/PPFD) was not recorded during the experiment, and direct photosynthetic and gas-exchange parameters were not measured. Therefore, the observed chlorophyll and stomatal responses should not be interpreted as direct evidence of photosynthetic performance. Future studies should incorporate direct measurements of soil and plant water status, greenhouse light intensity, and photosynthetic and gas-exchange parameters. Longer-term drought exposure, post-drought recovery following rewatering, root traits, and field-level growth and yield should also be evaluated.

## 4. Materials and Methods

### 4.1. Plant Materials and Growth Conditions

Eleven-month-old oil palm seedlings representing ten commercial genotypes used in Thailand were evaluated: Surat 2, PSU, Golden Tenera, Nong Ped, Univanich, Nigeria Black, CPI Hybrid, Deli Ghana, DLM Advance, and DLY Millennium. The seedlings were purchased directly from commercial suppliers and originated from registered nurseries with varietal certification. Varietal identity was based on supplier certification and was not independently verified using molecular markers. Genotype descriptions, including available parental backgrounds, were based on previous reports [31,32]. Most genotypes were derived from Deli-based crosses with different parental sources, including La Mé, AVROS, Yangambi, Ghana, and Nigeria Black, whereas CPI Hybrid was derived from Chumphon Dura × Chumphon Pisifera [31,32].

Seedlings were transplanted individually into 36-inch-diameter (91.4 cm) plastic pots containing the same commercially available soil-based potting medium containing coconut coir. The exact composition and physicochemical properties of the growing medium were not determined. Transplanting was completed within an approximately two-week period, after which the seedlings were maintained under the same greenhouse conditions and cultural practices prior to the initiation of the water treatments. All seedlings received the same fertilizer type, application rate, and fertilization schedule during this period and were 11 months old at the start of the experiment. However, complete uniformity in initial plant size among genotypes was not assumed because of inherent differences in growth characteristics among the commercial genotypes. Baseline measurements were therefore recorded for each individual plant at 0 days after treatment (DAT).

The experiment was conducted in a greenhouse in Nakhon Si Thammarat Province, Thailand, during June–July 2022 (approximately 8°28′ N, 99°56′ E). Air temperature ranged from 25 to 33 °C, and mean relative humidity was 80 ± 5%, as monitored using an Inkbird IBS-TH1 Plus data logger (Inkbird Tech Co., Ltd., Shenzhen, China). Light intensity (PAR/PPFD) was not recorded during the experimental period. Soil water potential was not measured during the experiment.

### 4.2. Experimental Design and Water-Withholding Treatment

Seedlings were acclimated for one month after transplanting and irrigated every 3–4 days to soil saturation. The experiment was arranged in a completely randomized design (CRD) with ten genotypes, two water regimes (well-watered and water-withholding), and three biological replicates (individual plants) per genotype × water-regime combination, resulting in a total of 60 plants. Pots were randomly positioned within the greenhouse at the beginning of the experiment and were not repositioned during the experimental period. Greenhouse position was not included as a blocking factor in the statistical analysis.

Following acclimation, well-watered plants were irrigated daily to drainage, whereas irrigation was completely withheld from plants assigned to the water-withholding treatment. Soil water content and plant water-status parameters were not measured during the experiment; therefore, the imposed treatment is more appropriately considered a water-withholding regime rather than a quantitatively defined level of soil or plant water deficit.

The water-withholding treatment was conducted for 45 days. Quantitative measurements were performed at 0, 15, and 30 days after treatment (DAT), with 0–30 DAT representing the primary quantitative assessment period. By 30 DAT, severe wilting prevented reliable measurement of some traits in some plants; these unavailable measurements were treated as missing values rather than biological zeros and were not imputed. At 45 DAT, severe wilting and tissue deterioration prevented reliable quantitative assessment, and observations and photographs were therefore retained only as qualitative documentation of the severe-injury endpoint.

### 4.3. Morphological and Vegetative Growth Traits

CW was measured at the widest point of each plant using consistent measurement positions throughout the experiment. FN was recorded as the total number of fully expanded fronds per plant at 15-day intervals.

At each sampling time, the third frond from the top was selected according to its relative position on the plant rather than by tracking the same individual frond over time. LLN and leaflet dimensions were determined from this frond. LLN was recorded as the total number of leaflets on the third frond.

Leaf area of the third frond was estimated according to [33] as:

*A* = 0.55 × *n* × *L* × *W*

where *A* is the estimated area of the third frond, *n* is the total number of leaflets on the third frond, and *L* and *W* are the mean leaflet length and mid-width (m), respectively. Estimated total leaf area per plant (LA, m^2^ plant^−1^) was then calculated as:

*LA* = *A* × *FN* = 0.55 × *n* × *L × W* × *FN*

### 4.4. Stomatal and Anatomical Traits

Three leaflets from the third frond from the top of each plant were collected at 15-day intervals and used for stomatal and anatomical observations. For stomatal observations, the cuticle was removed from the abaxial surface, stained with safranin O, mounted on microscope slides, and examined under a light microscope at 200× and 400× magnifications.

Stomata was counted at 400× magnification within a microscopic field area of 0.196 mm^2^ and converted to stomatal density (SN; stomata mm^−2^). SPW and SPL were measured between the inner boundaries of the guard cells, whereas GCW and GCL were measured across the outer boundaries of the guard-cell pair. Measurements were obtained from captured images enlarged fivefold and were initially recorded in centimeters. Values were converted to micrometres using the image calibration, whereby 0.5 cm on the enlarged image corresponded to 0.01 mm (10 µm) of the actual specimen; thus, 1 cm on the enlarged image corresponded to 20 µm. Twenty randomly selected stomata and their corresponding guard cells were measured for each biological replicate (individual plant). These measurements were treated as technical subsamples and averaged to obtain a single plant-level value for each trait at each sampling time. SPSI and GCPSI were calculated as SPW × SPL and GCW × GCL, respectively, and expressed in µm^2^. The plant-level means were used in all statistical analyses.

For anatomical observations, transverse leaflet sections were stained with 0.4% safranin O and examined for changes in bulliform cell morphology using a light microscope (Olympus CX23, Olympus Corp., Tokyo, Japan) equipped with an Olympus DP72 digital camera (Olympus Corp., Tokyo, Japan). Bulliform cell morphology was qualitatively examined in all ten genotypes at 0, 15, and 30 DAT. Four representative genotypes under the water-withholding treatment are presented in Figure 2, while observations for all ten genotypes under both water regimes are provided in Figure S1. No quantitative measurements or statistical analyses were performed for bulliform cell morphology.

### 4.5. Biochemical Analysis

Chlorophyll content was determined according to [34]. Fresh leaf samples (0.1 g) from the third frond were homogenized in 10 mL of hexane:ethanol (6:4, *v*/*v*) and centrifuged at 4000 rpm for 15 min at room temperature. Absorbance of the supernatant was measured at 645 and 663 nm using a UV–Vis spectrophotometer (UV-2600, Shimadzu, Kyoto, Japan), with hexane:ethanol (6:4, *v*/*v*) as the blank. Chl *a* and Chl *b* concentrations in the extract were calculated as follows:

Chl *a* (mg/100 mL extract) = 0.999A_663_ − 0.0989A_645_

Chl *b* (mg/100 mL extract) = −0.328A_663_ + 1.77A_645_

Chlorophyll contents were expressed on a fresh-weight basis (mg g^−1^ FW). Because 0.1 g of fresh tissue was extracted in 10 mL of solvent, the numerical values obtained in mg/100 mL extract were equivalent to those expressed in mg g^−1^ FW. TC was calculated as the sum of Chl *a* and Chl *b*.

### 4.6. Soluble Sugar and Starch Analyses

SC was determined using a modified anthrone method based on [35]. Leaf samples (0.4 g) from the third frond were homogenized in 10 mL of 80% ethanol and centrifuged at 4000 rpm for 20 min at room temperature. An aliquot (500 µL) of the supernatant was mixed with 5 mL of anthrone reagent, heated in boiling water for 10 min, and cooled in an ice bath for 10 min. Absorbance was measured at 620 nm using a UV–Vis spectrophotometer (UV-2600, Shimadzu, Kyoto, Japan). SC was determined from a glucose standard curve and expressed as mg L^−1^. The residual pellet was dried at 60 °C for 48 h and used for starch determination.

ST was determined using a modified anthrone method based on [36]. The dried residual pellet was weighed, suspended in 2.5 mL of distilled water and heated at 100 °C for 15 min. After addition of 1.5 mL of perchloric acid, the mixture was stirred at 5 min intervals for 15 min, diluted with 8 mL of distilled water, and centrifuged at 4000 rpm for 15 min. An aliquot (2.5 mL) of the supernatant was diluted to 25 mL with distilled water, and 2.5 mL of the diluted extract was mixed with 5 mL of anthrone reagent, heated at 100 °C for 7–10 min, and cooled in an ice bath for 10 min. Absorbance was measured at 630 nm using the same spectrophotometer. ST was determined from a glucose standard curve, adjusted using a glucose-to-starch conversion factor, and expressed on the basis of the dry weight of the residual pellet used for extraction (mg g^−1^ DW).

### 4.7. Statistical Analysis

Data were analyzed using R software (version 4.2.1; R Core Team, R Foundation for Statistical Computing, Vienna, Austria). Quantitative analyses were based on measurements at 0, 15, and 30 days after treatment (DAT); observations at 45 DAT were retained only as a qualitative endpoint. Temporal responses of morphological, stomatal, and biochemical traits were analyzed using split-plot/repeated-measures ANOVA, with genotype and water regime as between-subject factors and time as the within-subject factor. Individual plants were treated as subjects nested within each G × W combination, and all main effects and interactions were evaluated.

Model assumptions were assessed using the Shapiro–Wilk test for residual normality, Levene’s test for homogeneity of variance, and Mauchly’s test for sphericity. When sphericity was violated, Greenhouse–Geisser-adjusted degrees of freedom were applied. Some departures from residual normality were detected, but Greenhouse–Geisser correction did not alter the significance interpretation of time-related effects.

Changes from baseline to 30 DAT (Δ30–0) were analyzed by two-way ANOVA with G, W, and G × W as fixed effects, followed by Duncan’s Multiple Range Test (DMRT) at *p* < 0.05. Measurements unavailable because of severe wilting were treated as missing and were not imputed.

Pearson correlations among Δ30–0 values of CW, FN, LLN, LA, TC, SC, ST, SN, SPSI, and GCPSI were calculated separately for each water regime using individual-plant values and pairwise complete observations. The 45 pairwise *p*-values within each regime were adjusted using the Benjamini–Hochberg false-discovery-rate (FDR) procedure, with significance determined at FDR-adjusted *p* < 0.05.

For genotype-level multivariate analyses, relative response to water withholding at 30 DAT was calculated as (mean water-withholding value at 30 DAT/mean well-watered value at 30 DAT) × 100. PCA and HCA were restricted to the five traits with complete data across all ten genotypes (CW, FN, TC, SC, and ST) and were performed on Z-score-standardized relative responses. HCA used Euclidean distance and Ward’s minimum-variance method.

## 5. Conclusions

This study demonstrated substantial genotypic variation in the responses of 11-month-old oil palm seedlings to water withholding. Vegetative growth, stomatal characteristics, bulliform cell morphology, chlorophyll, and carbohydrate-related traits showed contrasting patterns among the ten commercial genotypes, while correlation and multivariate analyses revealed coordinated response patterns across these traits. HCA distinguished three groups based on standardized relative responses at 30 DAT for five traits with complete genotype-level data (CW, FN, TC, SC, and ST), representing contrasting multivariate response profiles under the imposed water-withholding treatment rather than definitive categories of drought tolerance. The findings indicate that multiple morphological, stomatal, and biochemical traits have potential utility for characterizing drought-response patterns in young oil palm. Integrating these complementary traits may provide a useful framework for early differentiation of contrasting commercial germplasm for subsequent drought evaluation and breeding. However, further validation using direct measurements of soil and plant water status, recovery responses following rewatering, and longer-term and field-based drought conditions is required before these traits can be used as definitive indicators of drought tolerance.

## Figures and Tables

**Table 1:** Analysis of variance (ANOVA) mean squares for morphological and vegetative growth traits of ten oil palm genotypes under well-watered and water-withholding treatments.

Source of Variation	df	Canopy Width (CW)	Frond Number (FN)	Leaflet Number (LLN)	Leaf Area (LA)
Genotype (G)	9	3784.8538 ***	24.3827 ***	341.5160 ***	2.3994 ***
Water regime (W)	1	15918.9031 ***	369.8000 ***	897.8000 ***	21.1077 ***
G × W	9	777.2287 ns	16.3679 ***	42.6642 ns	0.5766 **
Subject within G × W	40	719.4635	4.0611	21.2444	0.1511
Time (DAT)	2	1818.1218 ***	75.0500 ***	330.4167 ***	1.6027 ***
G × Time	18	547.9165 ***	7.7846 ***	66.8179 ***	0.2137 ***
W × Time	2	15724.3198 ***	179.4500 ***	1059.4500 ***	9.3202 ***
G × W × Time	18	385.9695 ***	10.5549 ***	13.6660 ns	0.2457 ***
Time × Subject within G × W	80	129.8268	0.7611	18.7694	0.0615

Data from 0, 15, and 30 DAT were analyzed using split-plot/repeated-measures ANOVA. Mean squares are shown. **, *p* < 0.01; ***, *p* < 0.001; ns, not significant. DAT, days after treatment.

**Table 2:** Analysis of variance (ANOVA) mean squares for stomatal and guard-cell traits of ten oil palm genotypes under well-watered and water-deficit treatments at 0, 15, and 30 days after treatment.

Source of Variation	df	Stomatal Pore Width (SPW)	Stomatal Pore Length (SPL)	Guard-Cell Width (GCW)	Guard-Cell Length (GCL)	Stomatal Number (SN)
Genotype (G)	9	203.2400 ***	848.0000 ***	2184.0400 ***	1679.5600 ***	131,899.1488 ***
Water regime (W)	1	235.7600 ***	785.4400 ***	2191.0400 ***	7194.6800 ***	85,520.0672 ***
G × W	9	16.6000 ***	63.1200 ***	169.9200 ***	146.3600 ***	4343.7578 ***
Subject within G × W	40	2.4800	14.6000	8.1200	3.5200	123.9353
Time (DAT)	2	116.9600 ***	963.6400 ***	2291.4800 ***	4019.3600 ***	63,427.1319 ***
G × Time	18	3.1200 ***	51.0800 ***	164.2400 ***	164.4800 ***	4065.9335 ***
W × Time	2	173.6400 ***	1676.2800 ***	2680.6800 ***	4724.6800 ***	66,268.5392 ***
G × W × Time	18	4.4800 ***	63.7600 ***	173.4400 ***	151.8800 ***	3845.0255 ***
Time × Subject within G × W	80	1.0800	5.9600	3.1600	2.6800	49.0967

Data from 0, 15, and 30 DAT were analyzed using split-plot/repeated-measures ANOVA. Mean squares are shown. ***, *p* < 0.001. SPW, stomatal pore width (µm); SPL, stomatal pore length (µm); GCW, guard-cell width (µm); GCL, guard-cell length (µm); SN, stomatal density (stomata mm^−2^); DAT, days after treatment.

**Table 3:** Analysis of variance (ANOVA) mean squares for biochemical traits of ten oil palm genotypes under well-watered and water-withholding treatments at 0, 15, and 30 days after treatment.

Source of Variation	df	Chlorophyll *a* (Chl *a*)	Chlorophyll *b* (Chl *b*)	Total Chlorophyll (TC)	Soluble Sugar Content (SC)	Starch Content (ST)
Genotype (G)	9	0.755582 ***	0.271553 ***	0.759547 ***	0.00397156 ***	0.0076314 ***
Water regime (W)	1	0.00443633 ns	0.0839729 ns	0.0498071 ns	0.000240156 *	0.00242281 ***
G × W	9	0.110317 **	0.273686 ***	0.515741 ***	3.30313 × 10^−5^ ns	0.00120689 ***
Subject within G × W	40	0.0378203	0.056905	0.115913	4.14824 × 10^−5^	8.61787 × 10^−5^
Time (DAT)	2	0.111468 ns	0.388001 ***	0.79734 **	2.32126 ***	0.0169329 ***
G × Time	18	0.132415 ***	0.108699 **	0.184231 ns	0.000989309 ***	0.00383328 ***
W × Time	2	0.194741 **	0.0361447 ns	0.260818 ns	1.56024 × 10^−5^ ns	0.000406569 ns
G × W × Time	18	0.156519 ***	0.177973 ***	0.263891 **	4.07973 × 10^−5^ ns	0.000414991 ns
Time × Subject within G × W	80	0.0388709	0.0426282	0.107417	3.67754 × 10^−5^	0.000261407

Data from 0, 15, and 30 DAT were analyzed using split-plot/repeated-measures ANOVA. Mean squares are shown. G, genotype; W, water regime; DAT, days after treatment. *, *p* < 0.05; **, *p* < 0.01; ***, *p* < 0.001; ns, not significant.

## Data Availability

The data presented in this study are available within the article and Supplementary Materials. Additional data are available from the corresponding author upon reasonable request.

## Supplementary Materials

The following supporting information can be downloaded at: https://www.mdpi.com/article/10.3390/plants15203165/s1. Figure S1: Bulliform cell morphology of ten oil palm genotypes under well-watered and water-withholding conditions at 0, 15, and 30 days after treatment (DAT); Table S1: Temporal changes in morphological and vegetative growth traits of ten oil palm genotypes under well-watered and water-withholding conditions at 0, 15, and 30 days after treatment (DAT); Table S2: Two-way analysis of variance (ANOVA) of changes from 0 to 30 days after treatment (Δ30–0) in morphological, stomatal, and biochemical traits of ten oil palm genotypes under well-watered and water-withholding conditions; Table S3: Baseline (0 DAT) absolute values of biochemical and stomatal traits of ten oil palm genotypes under the two assigned water-regime groups; Table S4: Changes in morphological, biochemical, and stomatal traits of ten oil palm genotypes from 0 to 30 days after treatment (Δ30–0) under well-watered and water-withholding conditions; Table S5: Complete Pearson correlations among changes from 0 to 30 DAT (Δ30–0) under well-watered and water-withholding conditions, with Benjamini–Hochberg false-discovery-rate (FDR) adjustment; Table S6: Principal component analysis (PCA) of Z-score-standardized relative responses to water withholding at 30 days after treatment (DAT); Table S7: Hierarchical cluster membership based on Z-score-standardized relative responses to water withholding at 30 days after treatment (DAT).

## Abbreviations

The following abbreviations are used in this manuscript:

ANOVA	analysis of variance
Chl *a*	Chlorophyll *a*
Chl *b*	chlorophyll *b*
CRD	completely randomized design
CW	canopy width
DAT	days after treatment
FDR	false discovery rate
FN	frond number
G	genotype
GCL	guard-cell length
GCPSI	guard-cell pair size index
GCW	guard-cell width
HCA	hierarchical cluster analysis
LA	leaf area
LLN	leaflet number
PCA	principal component analysis
PC1	first principal component
PC2	second principal component
SC	soluble sugar content
SN	stomatal density
SPL	stomatal pore length
ST	starch content
SPW	stomatal width
SPSI	stomatal pore size index
T	time
TC	total chlorophyll
W	water regime
