# Supplementary material for: Integrated Drought-Response Profiles Reveal Genotypic Variation Among Commercial Oil Palm (Elaeis guineensis Jacq.) Genotypes
Source: Plants (Basel). 2026 Oct 16;15(20):3165. doi: 10.3390/plants15203165 (PMC13709227; doi:10.3390/plants15203165)
Supplement: Supplementary file 1 [file plants-15-03165-s001.zip › Figure S1. Bulliform cell morphology of ten oil palm genotypes.pdf]

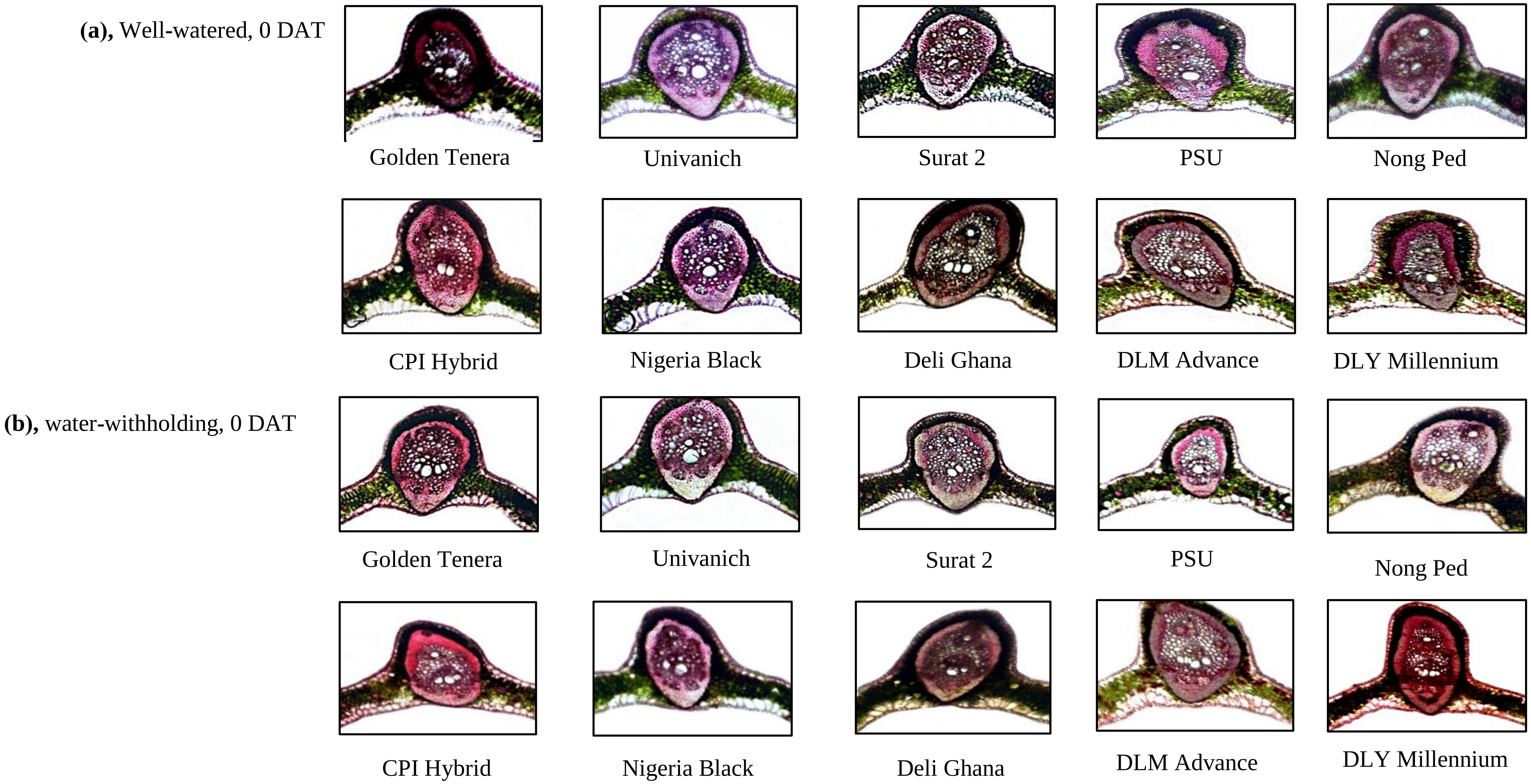
(a), Well-watered, 0 DAT
Golden Tenera
Univanich
Surat 2
PSU
Nong Ped
CPI Hybrid
Nigeria Black
Deli Ghana
DLM Advance
DLY Millennium
(b), water-withholding, 0 DAT
Golden Tenera
Univanich
Surat 2
PSU
Nong Ped
CPI Hybrid
Nigeria Black
Deli Ghana
DLM Advance
DLY Millennium

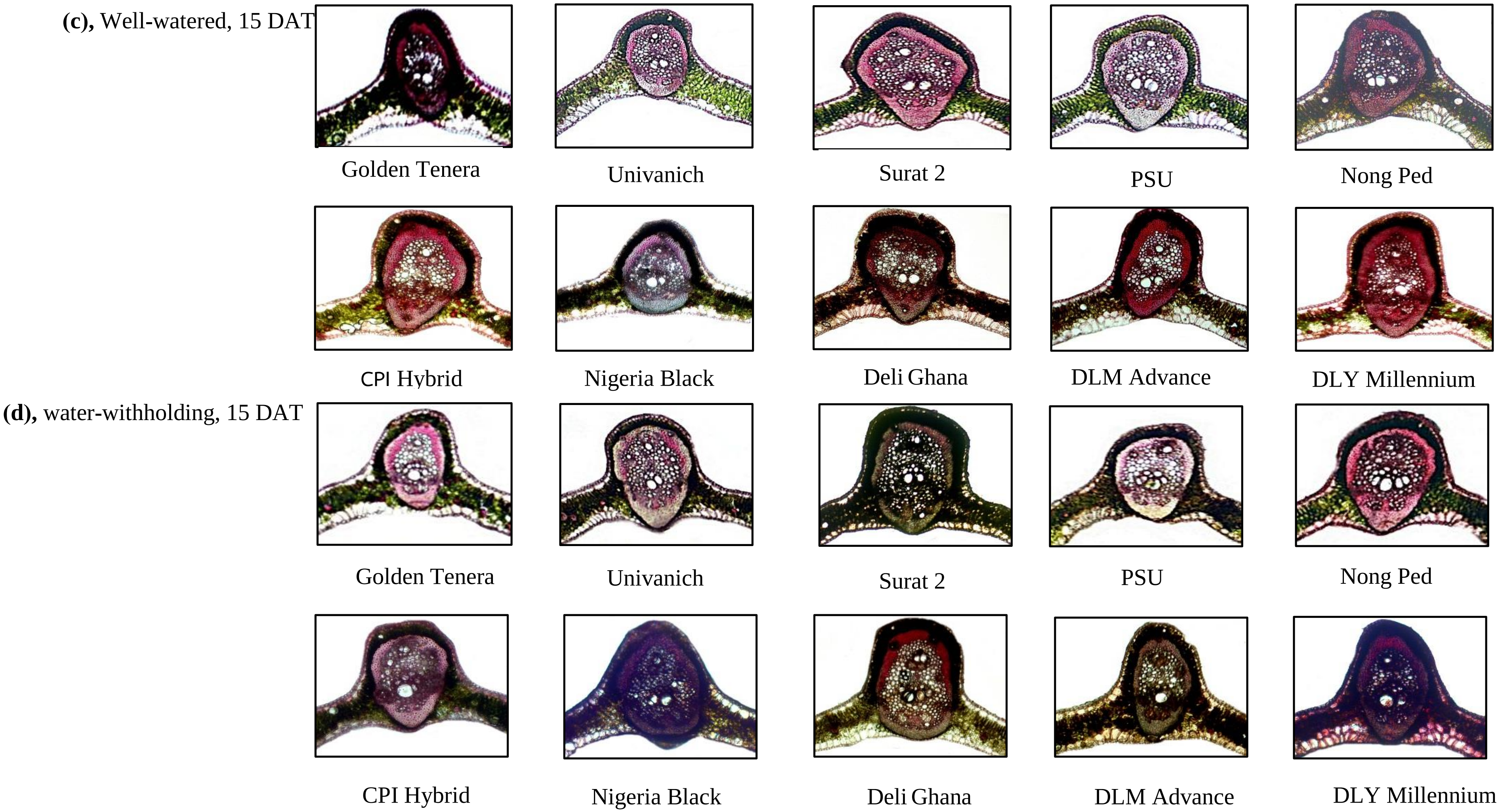

(c), Well-watered, 15 DAT
Golden Tenera
Univanich
Surat 2
PSU
Nong Ped
CPI Hybrid
Nigeria Black
Deli Ghana
DLM Advance
DLY Millennium
(d), water-withholding, 15 DAT
Golden Tenera
Univanich
Surat 2
PSU
Nong Ped
CPI Hybrid
Nigeria Black
Deli Ghana
DLM Advance
DLY Millennium

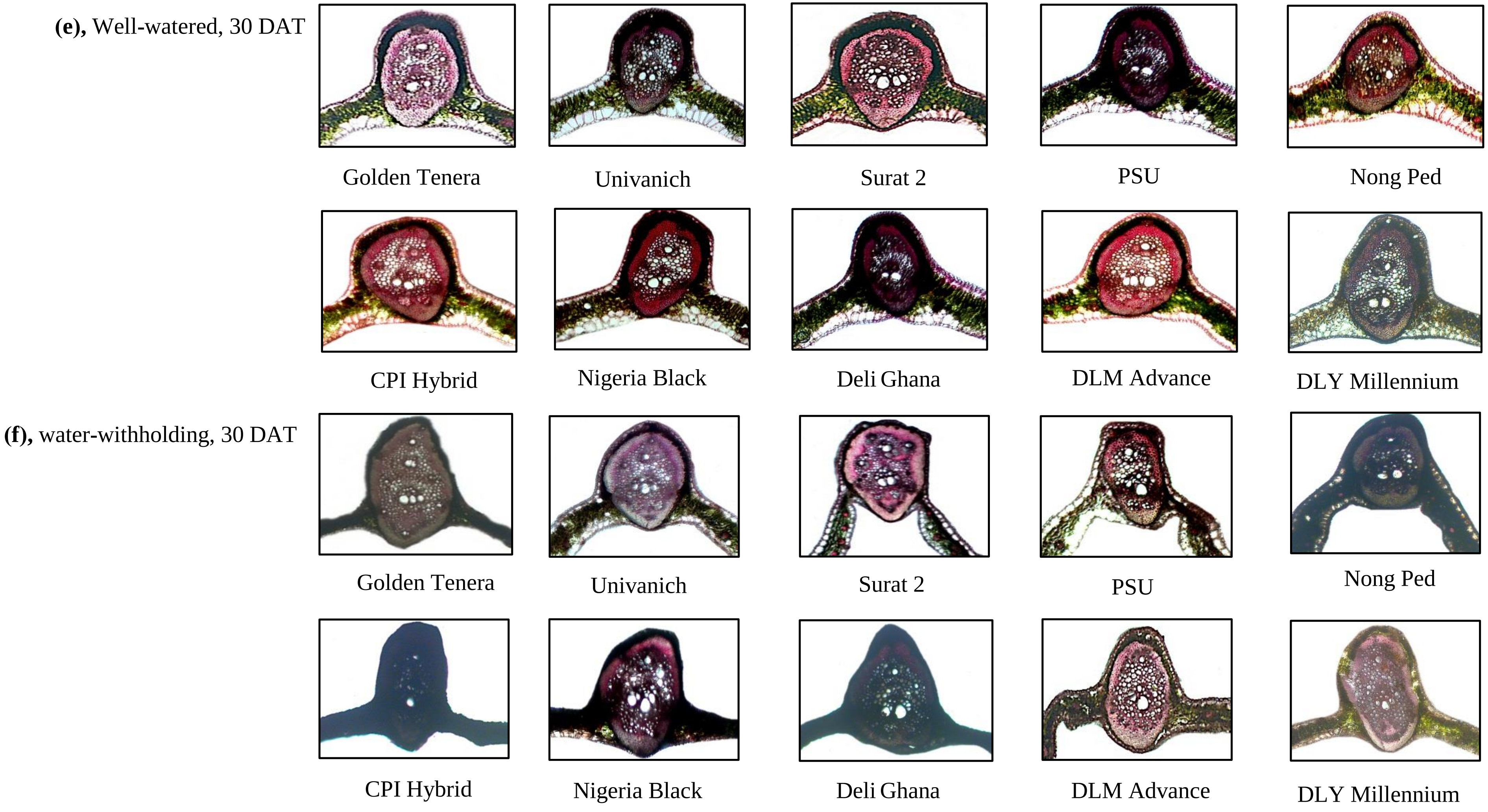

Figure S1. Bulliform cell morphology of ten oil palm genotypes under well-watered and water-withholding conditions at 0, 15, and 30 days after treatment (DAT): (a) well-watered, 0 DAT; (b) water-withholding, 0 DAT; (c) well-watered, 15 DAT; (d) water-withholding, 15 DAT; (e) well-watered, 30 DAT; and (f) water-withholding, 30 DAT. Images are presented in the same genotype order across all panels to facilitate temporal comparison. All images were captured at the same magnification (5×).
